# Analysis of volatile organic compounds in exhaled breath to diagnose ventilator-associated pneumonia

**DOI:** 10.1038/srep17179

**Published:** 2015-11-26

**Authors:** Ronny Schnabel, Rianne Fijten, Agnieszka Smolinska, Jan Dallinga, Marie-Louise Boumans, Ellen Stobberingh, Agnes Boots, Paul Roekaerts, Dennis Bergmans, Frederik Jan van Schooten

**Affiliations:** 1 Department of Intensive Care Medicine, Maastricht University Medical Centre+, Maastricht, the Netherlands; 2 Department of Pharmacology and Toxicology, School for Nutrition and Translational Research in Metabolism (NUTRIM), Maastricht University Medical Centre+, Maastricht, The Netherlands; 3 Department of Medical Microbiology, Maastricht University Medical Centre+, Maastricht, the Netherlands

## Abstract

Ventilator-associated pneumonia (VAP) is a nosocomial infection occurring in the intensive care unit (ICU). The diagnostic standard is based on clinical criteria and bronchoalveolar lavage (BAL). Exhaled breath analysis is a promising non-invasive method for rapid diagnosis of diseases and contains volatile organic compounds (VOCs) that can differentiate diseased from healthy individuals. The aim of this study was to determine whether analysis of VOCs in exhaled breath can be used as a non-invasive monitoring tool for VAP. One hundred critically ill patients with clinical suspicion of VAP underwent BAL. Before BAL, exhaled air samples were collected and analysed by gas chromatography time-of-flight mass spectrometry (GC-*tof-*MS). The clinical suspicion of VAP was confirmed by BAL diagnostic criteria in 32 patients [VAP(+)] and rejected in 68 patients [VAP(−)]. Multivariate statistical comparison of VOC profiles between VAP(+) and VAP(−) revealed a subset of 12 VOCs that correctly discriminated between those two patient groups with a sensitivity and specificity of 75.8% ± 13.5% and 73.0% ± 11.8%, respectively. These results suggest that detection of VAP in ICU patients is possible by examining exhaled breath, enabling a simple, safe and non-invasive approach that could diminish diagnostic burden of VAP.

Ventilator-associated pneumonia (VAP) is a common hospital-acquired infection occurring in the intensive care unit (ICU) with an incidence that varies from 4–42% depending on the applied diagnostic criteria^1^. It is a severe complication of mechanical ventilation with an attributable mortality risk of approximately 13%^2^. To date, the diagnosis is based on clinical criteria in combination with bacterial culture results. In patients clinically suspected of having VAP, bronchoalveolar lavage (BAL) from the site of the presumed infection and subsequent cytological and microbiological analysis of the lavage fluid is regarded a suitable diagnostic approach^3^. However, this technique is invasive, involves risks and has its limitations in patients with severe pulmonary disease, high respiratory support settings and coagulation abnormalities. Additionally, analysis of BAL is laborious, time- consuming and takes up to 48 hours before definitive results are available. Only then can the diagnosis of VAP be confirmed or rejected. During this period patients empirically receive broad spectrum antibiotics. Facing a rapid emergence and dissemination of multi-drug resistant microorganisms particularly in the ICU environment, strategies to reduce such general and non-targeted antibiotic consumption have become very important^4^.

It is therefore of interest to find a new method that allows fast, reliable, non-invasive VAP diagnosis. Using exhaled breath for disease diagnosis is a promising technique that may be able to fulfil these criteria. Exhaled breath contains a multitude of volatile organic compounds (VOCs) originating from both exogenous and endogenous sources. Endogenous VOCs are produced by biological processes including oxidative stress and inflammation in the human body^5^,^6^ as well as by invading microorganisms^7^. Upon their production, VOCs are excreted into the blood after which they diffuse into the lungs where they are exhaled. Oxidative stress and inflammation induce alterations in the composition of VOCs excreted by the affected organ and thus the exhaled breath. Additionally, microorganisms themselves may produce specific compounds leading to different VOC profiles in exhaled breath. Taking into account the invasion of harmful microorganisms in the lungs and the defence mechanisms that are subsequently set in motion by the host, it can be expected that VOCs are present in different concentrations and compositions in patients with VAP compared to patients without VAP. These discriminating VOC profiles may be used to aid VAP diagnosis.

Thus far, discriminating VOC profiles have been found for various respiratory diseases such as chronic obstructive pulmonary disease (COPD), asthma, tuberculosis and cystic fibrosis^8^,^9^,^10^,^11^,^12^,^13^. It has already been demonstrated that *Pseudomonas aeruginosa*, *Staphylococcus aureus*, *Escherichia coli* and *Klebsiella pneumoniae* could be identified correctly based on the analysis of VOCs excreted into the headspace of cultured bacteria^14^. Many of these strains frequently cause VAP. In another study, VOCs of *Streptococcus pneumoniae* and *Haemophilus influenzae* cultures were analysed at different time points during cultivation, leading to the identification of strain-specific VOCs for both bacterial species^15^. A systematic review summarized both strain-specific and commonly occurring VOCs from 31 recent *in vitro* studies that investigated bacterial species^7^. A recently published study by Fowler *et al.* found that, in a well-characterized group of patients with sterile brain injury, exhaled breath analysis can adequately detect the presence of airway pathogens *in vivo* that can induce VAP^16^. The aim of the current study is to identify VAP-specific VOCs *in vivo* by analysis of the exhaled breath of critically ill mechanically ventilated patients independent of their underlying disease upon admission to the ICU.

## Materials and Methods

### Study design

This study was conducted at Maastricht University Medical Centre + , a tertiary, university hospital in the Netherlands with 1,700 ICU admissions per year. The ICU consists of two 9- bed units for medical and surgical patients and one 9-bed unit for cardiothoracic surgery patients. Adult critically ill, mechanically ventilated patients with a clinical suspicion of VAP who underwent a diagnostic BAL were included. Exclusion criteria for the BAL procedure were thrombocytopenia (<40,000/μL) and other coagulation abnormalities. The exhaled breath study and its experimental protocols were evaluated by the joint medical ethics committee at Maastricht University and Maastricht University Medical Centre + (METC azM/UM). After evaluation and approval of the experimental protocols, the METC azM/UM committee concluded that the study did not fall under the scope of the medical research involving human subjects act (WMO), and was therefore denoted as “non-WMO research” as no direct and invasive patient intervention was required and results of the analyses did not influence the patient’s outcome. Experimental protocols were performed in accordance with the approved national Dutch guidelines for non-WMO research^17^.

A patient was clinically suspected of VAP after ≥ 48 hours of mechanical ventilation, fulfilling the clinical criteria depicted in Table 1. BAL was performed on the day of clinical suspicion for VAP. A fibreoptic bronchoscope (Pentax FB-15 H/FB-15 X, Pentax Medicals, Tokyo, Japan) was introduced and ‘wedged’ into the affected segmental or subsegmental bronchus. Sterile saline (0.9% sodium chloride at room temperature) was instilled in four aliquots of 50 mL, immediately aspirated and recovered. Further analysis was highly standardized as described by de Brauwer *et al.*^18^. A clinically suspected episode was considered microbiologically confirmed when the following criteria were met in BAL fluid (BALF): presence of ≥2% cells containing intracellular organisms (ICO) and/or quantitative culture results of ≥10^4^ cfu/mL^19^,^20^. One hundred patients were included in the study. Upon BALF analysis they were divided into two groups: (1) BALF confirmed the clinical suspicion of VAP (VAP(+), n = 32); (2) the diagnosis of VAP was rejected by BALF analysis (VAP(−), n = 68). The *Sequential Organ Failure Assessment* (SOFA) score was registered at the moment of BAL to compare the seriousness of illness. The diagnosis of the underlying disease on admission to the ICU of all patients were documented and allocated into seven diagnostic groups. Differences between VAP(+) and VAP(−) were tested for significance: two-sided paired t-test for age and SOFA scores; chi square for diagnosis upon admission. A p-value < 0.05 was considered significant. (Table 2)

### Sampling and measurement of exhaled breath

Directly before BAL was performed, exhaled breath samples from ventilated patients were collected into a sterile Tedlar bag (5 L). The bag was tightly connected to the expiratory limb of the Draeger® Evita XL ventilator (Lübeck, Germany). Exhaled breath from the patient could then flow into the Tedlar bag without any pollution from the environment. When the bag was filled, its valve was closed and the connection with the ventilator subsequently removed. The content of the bag was transported by vacuum pump (VWR International, France) onto stainless steel two-bed desorption tubes filled with carbograph 1TD/Carbopack X (Markes International, Llantrisant, Wales, UK) that trap VOCs. The VOCs captured in these desorption tubes were measured by gas chromatography-*time of flight-*mass spectrometry (GC-*tof*-MS) based on the procedure described by Van Berkel *et al.*^8^. This was done in a non-targeted way, meaning that the highest amount and variety of VOCs were measured and used for multivariate statistical analysis later on.

### Data processing and statistical analysis

Raw GC-*tof*-MS data were pre-processed to remove various sources of artefacts before the actual statistical analysis. Pre-processing of the data reduces the influence of these artefacts and allows for the biological variation to come through. This was done by sequential use of the following methods: denoising, baseline correction, alignment, normalization and scaling of the data^21^. In order to compare different groups, the number of samples in the larger group has to be reduced to the size of the smaller group to make the statistical analyses work efficiently. This was done by randomly choosing a subset of 32 samples of the 68 VAP(−) samples to match the size of the VAP(+) group and using this subset for further statistical analysis. This procedure was repeated 250 times to ensure that each sample in the larger group was used. For this study, the multivariate statistical analysis method Random Forest (RF) was used^22^. This machine learning method constructs a multitude of de-correlated decision trees to classify samples into the appropriate disease state. Decision trees are predictive models that try to classify samples based on a specific subset of the measured VOCs. RF creates many decision trees (e.g. 1,000) comprising of a small and randomly selected subset of VOCs and tries to predict the class outcome. The most discriminatory subset of VOCs is then used to create the final classification model. Validation of the RF model was done by calculating the “out-of-bag error”. In this procedure 66.7% of the samples are randomly selected with replacement for each decision tree. The remaining 33.3% are used to calculate the performance of the RF classification model. This produces class probability values, which are used to calculate sensitivity and specificity illustrated by Receiver Operating Characteristic (ROC) curves. For the sensitivity and specificity parameters, the 95% confidence interval was calculated and written in the following way: mean ± confidence interval. A ROC curve is a graphical representation of the performance of the predictive model established by RF. The area under the curve (AUC) is most commonly used as an indicator of predictive performance: a value close to 1 indicates high predictive power of the model, whereas an AUC close to 0.5 means that the model has no predictive power^23^.

For visualization purposes, principal component analysis (PCA) score plots of the RF proximities were created. The proximities are distance parameters ranging from 0 to 1 that visualize similarities of the selected VOC profile between individual samples. A small proximity value indicates similarity, while a large proximity value indicates dissimilarity between individuals. A PCA plot of proximities can therefore demonstrate groupings of samples and trends in the data.

### Influence of confounders

To rule out that the VOC profile found by RF is influenced by confounding factors, regularized MANOVA was used^24^ to test for the following possible confounders: age, gender, diagnostic group at admission, SOFA scores, ICU mortality, general hospital mortality, the presence of comorbidities in general, and the presence of specific comorbidities mentioned in Table 2.

### Compound identification

The VOCs implemented into the classification model were identified with spectrum recognition using the National Institute of Standard and Technology (NIST) library in combination with spectrum interpretation by an experienced mass spectrometrist and identification based on retention times of components.

### Pathway identification

For each of the chemically identified VOCs the ChEBI^25^, ChemSpider^26^ and PubChem IDs^27^ were found in their respective databases. BridgeDb^28^, which links identifiers from several databases, was used to find additional identifiers corresponding to each VOC. This was necessary due to annotation problems in pathways, where the same metabolite is mentioned with a different identifier or name in different pathways.

The RRDF package^29^ was then used to find pathways in Wikipathways^30^ that included that specific VOC. Additionally, because Wikipathways includes only a limited number of metabolite pathways, the KEGG database^31^ was analysed for pathways containing the identified VOCs. This was done using the KEGG REST api.

Because VAP is mainly caused by a well-defined array of bacteria, a selection of pathways was made that were present in the human host or in bacteria most likely to cause VAP^32^ including *Staphylococcus aureus*, *Pseudomonas aeruginosa*, *Escherichia coli*, *Klebsiella pneumonia*e, *Hemophilus influenzae*.

## Results

### Clinical outcome/data

An overview of demographic and clinical data is presented in Table 2. Sample size varies between the groups as a result of confirmation of VAP in only 32 of 100 individuals included in the study. There were no significant differences in the seriousness of disease at the moment of BAL (SOFA) or in the distribution of the underlying diagnosis on admission to the ICU. However, patients in the VAP(−) group seemed to suffer more from a haematological diagnosis upon admission and active malignancies. (Table 2) During the study period we found an average of 2.5 episodes of VAP per 1,000 ventilator days. The diagnosis of VAP was based in 6 patients on a percentage of ICO > 2% alone and in 26 patients on a bacterial growth of more than 10^4^ cfu/mL. *Staphylococcus aureus* (n = 5), *Pseudomonas aeruginosa* (n = 4), *Escherichia coli* (n = 4), *Klebsiella pneumonia*e (n = 4), *Hemophilus influenzae* (n = 3) and *Acinetobacter baumannii* (n = 3) were the most frequently found microorganisms.

### RF classification model

**Figure 1:**
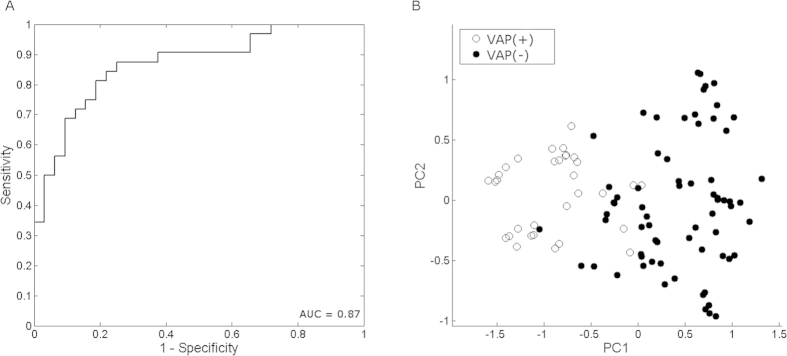
ROC and PCA plots visualizing the separation of the VAP(+) and VAP(−) groups. (**A**) Receiver operating characteristic (ROC) curve of VAP(+) vs. VAP(−). It consists of 1-sensitivity on the x-axis and specificity on the y-axis. (**B**) The PCA plot is based on the proximities between samples of VAP(+) (white) and VAP(−) (black).

GC-MS measurements produced 100 chromatograms: one for each patient. After processing, these chromatograms consisted of >1000 chemically different VOCs. RF was used to filter VOCs that were discriminatory between VAP(+) and VAP(−). The final RF classification model was based on 12 discriminatory VOCs and correctly classified 74.2% ± 13.8% of all individuals with a sensitivity and specificity of 75.8% ± 13.5% and 73.0% ± 11.8% respectively. The corresponding ROC curve depicted in Fig. 1A had an AUC of 0.87. The PCA score plot of proximities between the individual samples based on the 12 most important VOCs (Fig. 1B) showed that the VAP(+) and VAP(−) patients are separated with small overlap. This indicates that patients suffering from VAP can be identified based on this combination of 12 VOCs with high accuracy.

### Influence of confounders

The influence of potential confounders was tested to ensure that the discriminating VOC profile was purely a result of the VAP diagnosis. The following confounding factors were tested: age, gender, diagnostic group at admission, SOFA scores, ICU mortality, general hospital mortality, the presence of comorbidities in general, and the presence of specific comorbidities mentioned in Table 2. None of these confounders significantly influenced the model (Supplementary Table S1).

### Compound identification

The chemical identity of the 12 VOCs selected by RF is shown in Table 3. The identified VOCs include 2-methylbutane, heptane, dodecane and tetradecane (alkanes), carane (hydrocarbon ring structure), ethanol and isopropyl alcohol (alcohols), acrolein and tetradecanal (aldehydes). The remaining compounds were identified as acetone (ketone), ethylbenzene (aromatic hydrocarbon) and tetrahydrofuran (oxygen-containing heterocyclic compound).

### Pathway identification

The Wikipathways and KEGG databases were searched for pathways containing one or more of the 12 discriminatory VOCs. Only pathways present in humans and VAP-causing bacteria were included in the analysis. Seven KEGG pathways remained containing two VOCs (Table 4): one pathway that produced acrolein; five pathways with ethanol as an end-product and one which utilized ethanol. No human- or bacteria-specific Wikipathways were found.

## Discussion

In the present study, VOC profiles were determined in exhaled breath of patients clinically suspected of VAP to discriminate patients with VAP from other critically ill ventilated patients. Of 100 patients, 32 were diagnosed with VAP by quantitative BAL analysis. This ratio was in line with earlier publications^20^,^33^. A discriminating profile of 12 exhaled VOCs was identified that could determine the presence of VAP with an accuracy of 74.2% ± 13.8% , accompanied by a sensitivity of 75.8% ± 13.5%, a specificity of 73.0% ± 11.8% and an AUC of 0.87. The 12 VOCs that were identified by the model were chemically diverse. There was no significant difference in the diagnosis at admission or in the frequency and distribution of comorbidities between the VAP(+) and VAP(−) group of patients. However, a haematological diagnosis at admission and active malignancy as comorbidity were more prevalent among VAP(−) patients. Several potential confounders, including haematological diagnosis and active malignancies, were tested and proven not to be significantly associated with the VOC profile. The predominance of male patients in the demographics of the present study reflects known gender differences in the incidence of sepsis and VAP^34^,^35^.

These results demonstrate the potential of exhaled breath as a diagnostic tool in the ICU, where less invasive and faster detection methods are of great importance. Although the results are encouraging, the external validation in a large, multicentre cohort is necessary for clinical application. The advantage of exhaled breath over BAL analysis - the current gold standard for diagnosis of VAP - is that it is easy to perform, non-invasive and can be analysed within a short time span. In contrast to BAL, where the time to diagnosis is at least 48 h, exhaled breath sampling could take as little as one hour to get a diagnosis. To achieve this, exhaled breath, upon sampling after clinical suspicion of VAP, should be immediately transferred to a laboratory where it has to be processed by a mass spectrometer right away and subsequently checked for markers of VAP using a predesigned and validated algorithm. Eventually, such a fast diagnostic tool could support tailored antibiotic treatment, thereby aiding antibiotic resistance^4^. Additionally, it could reduce hospital costs and medication use^36^.

Thus far, most of the research done on exhaled breath in diagnosis of VAP was done with the e-nose technology. One study tested the use of e-nose technology as a substitute for chest computed tomography scan as a diagnostic tool for diagnosing VAP^37^. Although a prediction value of 80% was discovered, these results are difficult to interpret correctly due to the lack of independent validation. A more recent study by the same authors evaluated the use of the e-nose as a substitute for the Clinical Pulmonary Infection Score (CPIS)^38^. The CPIS is a measure of pulmonary infection that is used to diagnose VAP with an arbitrary cut-off of 6 for the diagnosis [ > 6 = VAP( + ), < 6 = VAP(−)]. However, since the CPIS is considered to be not reliable enough to diagnose VAP in the clinical setting^39^, it is unclear whether the included patients were accurately diagnosed with VAP, which could consequently skew their findings. Bos *et al.* also performed a prospective cohort study on diagnosing VAP with the e-nose^40^. They collected tracheal aspirates (TAs), and successively analysed the headspace of a bacterial culture medium of these TAs. They found an AUC of 0.85 in their cross-sectional study, which is comparable to our observed AUC of 0.87. All of these studies utilize the e-nose technology which has some limitations including reproducibility, negative effects of temperature and humidity, and the inability to identify the chemical identity of VOCs underlying the disease^41^. Knowing the identity of a VOC enables us to look at the underlying biological mechanisms of the disease.

Acute respiratory distress syndrome (ARDS) describes a condition of severe lung failure that can be caused by various non-infectious and infectious diseases including VAP. Additionally mechanical ventilation in patients with ARDS can facilitate the development of VAP. Exhaled breath as a diagnostic marker of ARDS was recently tested and could correctly classify ARDS patients and controls with an AUC of 0.78^42^. In our study, an AUC of 0.87 was found for VAP, which suggests that VAP may produce more pronounced differences in the exhaled breath. The ARDS study was performed using GC-MS and identified three VOCs to be essential for the discriminating model, of which two alkanes and one aldehyde. Although none of the individual compounds corresponded to the VOCs identified in our study, we found alkanes and aldehydes as well. This could imply involvement of similar underlying biological mechanisms in VAP and ARDS as well as sufficient differences between the pathology of the conditions to make them distinguishable by exhaled breath.

Over the last few years, multiple studies were performed to identify VOCs specific to a certain strain of bacteria. A recent review summarized all VOCs found for the six most frequently found bacteria in the ICU: *Staphylococcus aureus*, *Streptococcus pneumoniae*, *Enterococcus faecalis*, *Pseudomonas aeruginosa*, *Klebsiella pneuomoniae* and *Escherichia coli*^7^. These are all known to cause VAP in critically ill patients^43^. Another recent study detected VOCs that could differentiate various species of bacteria *in vitro*^14^. Although some of the bacteria included in this study were similar to those present in the current VAP patients, no overlap between the discriminating VOCs was observed. Aside from the small number of patients affected by each of these individual bacteria, this lack in overlap could also be explained by the fact that the *in vitro* study compared strains among one another whereas the present study compared diseased patients and healthy controls. More generally, all of the studies described here were performed *in vitro* or *ex vivo* whereas our study has been carried out with *in vivo* samples from a very heterogeneous patient population. This may also explain the discrepancies with our findings, as there are well-known limitations to the translatability of *in vitro* and *ex vivo* experiments into an *in vivo* situation^44^.

Recently a study was published by Fowler *et al.* where 46 ICU patients with sterile brain injury were followed as some of them developed a significant presence of airway pathogens (>10^4^ cfu/mL) that ultimately may have led to VAP in these patients^16^. Their exhaled breath was sampled and analysed in a similar manner as in our study using thermal desorption coupled with gas chromatography – time of flight – mass spectrometry, followed by multivariate analysis of the data. Likewise, BAL results were used as a reference in the diagnosis of VAP. Remarkably, 33% of the monitored patients demonstrated significant growth of pathogens in the lower respiratory tract. Hence, the incidence of VAP was much higher than in our ICU where we found an average of 2.5 episodes of VAP in 1,000 ventilator days^45^. This discrepancy might be explained by a higher risk of aspirations in patients with brain injury requiring intubation^46^. Although the patient population may not adequately reflect the overall ICU population, the results from Fowler *et al.* are very promising as a means to associate bacterial colonisation of the lower respiratory tract with exhaled VOCs. In contrast, our study reflects more the current clinical guidelines in the diagnosis of VAP, and the heterogeneity of an ICU population.

The list of VOCs found for VAP(+) vs. VAP(−) comparison consists of both endogenous and exogenous sources. Some endogenous compounds may be useful as they can indicate the cellular processes underlying VAP.

Ethanol is a compound that is produced by both bacteria and the human host. It is produced as a metabolite end-product in all but one of these pathways, which is reflected in the increased level of exhaled ethanol in VAP(+) patients.

Acetone is produced by spontaneous decarboxylation of acetoacetate, which is produced as a result of the build-up of ketone bodies. These ketones can be formed in the liver as a result of sepsis^47^. As a consequence of acetone build-up, isopropyl alcohol is formed as break-down product of acetone during ketogenesis. The exhaled concentrations of both compounds were lower in VAP(+) compared to VAP(−) patients. This can be explained by the fact that ketogenesis is reduced during inflammatory or infectious states^47^, resulting in less production of acetone and isopropyl alcohol.

Acrolein is also very likely to originate from endogenous sources and can be produced by a number of cellular processes. Firstly, lipid peroxidation accounts for a small portion of the endogenously produced acrolein. Secondly, myeloperoxidase (MPO) plays a crucial role in oxidative stress and the immune response to bacteria and oxidizes threonine into acrolein. Lastly, polyamines can also be catabolized into acrolein^48^. These polyamines are essential to the cell as they influence a range of processes from RNA and DNA structure to enzyme activity^49^. Additionally, one KEGG pathway was found where acrolein is formed as a breakdown product of anti-cancer drugs. A few patients in this study received cyclophosphamide, however the percentage of these patients did not differ between VAP(+) and VAP(−) group. It is therefore likely that the different abundance in acrolein originates from one of the described endogenous pathways.

Five of the 12 VOCs identified by the model can be classified as (branched) alkanes: heptane, 2-methylbutane, dodecane, tetradecane and tetradecanal. Two primary processes could account for the presence of alkanes in exhaled breath. First, lipid peroxidation as a result of oxidative stress is able to produce hydrocarbons. Heptane is thought to originate from oleic acid, and 2-methylbutane may originate from 2-methyl-1,3-butadiene (also known as isoprene). Second, alkanes are also present in the environment and are inhaled on a daily basis. After ingestion, the compounds are broken down in the liver by cytochrome P450 enzymes (CYP). The activity of these enzymes decreases with aging, but also with disease, implicating reduced CYP activity in severely ill patients^50^. Both mechanisms could explain the different abundances found between VAP(+) and VAP(−). Ethylbenzene is an benzene derivative and an indoor pollutant^51^. Benzene and its derivatives are also broken down in the liver by CYP enzymes, which may not function properly in critically ill patients, resulting in different exhaled abundances of ethylbenzene in VAP(+) vs. VAP(−) patients^52^. The two remaining compounds, tetrahydrofuran and carane, are also environmental pollutants and have no known endogenous source. Both compounds are likely metabolized by CYP enzymes in the liver, but no literature was found that supports this theory.

A limitation of the present study is the relatively small number of subjects. We were unable to test for specific strains of bacteria. As VAP is generally caused by an array of bacteria, we only had a few patients per bacterial strain available at most, hindering the use of multivariate statistics to identify strain-specific VOCs *in vivo*.

Although the quantitative culture analysis of BAL is accepted as state-of-the art in the diagnosis of VAP^53^, the sensitivity and specificity were variable among earlier histopathology studies with percentages of 42–93% and 45–100%, respectively^54^,^55^,^56^. This could have led to misdiagnosing several patients which could have possibly influenced the findings of the current study. However, the use of RF as multivariate technique reduces the influence of mislabelled samples on the outcome.

## Conclusion

The present study has demonstrated that it is possible to distinguish ICU patients with VAP from patients without VAP based on a profile of only 12 VOCs. Exhaled breath analysis is a promising, simple, safe and non-invasive technique for the rapid diagnosis of VAP. A larger study population is warranted to confirm our findings. Additionally, studies should be performed where strain-specific VOC profiles can be found.

### Key Messages

- Exhaled breath enables non-invasive diagnosis of Ventilator-Associated Pneumonia

## Additional Information

**How to cite this article**: Schnabel, R. *et al.* Analysis of volatile organic compounds in exhaled breath to diagnose ventilator-associated pneumonia. *Sci. Rep.* **5**, 17179; doi: 10.1038/srep17179 (2015).

## Supplementary Material

Supplementary Information

## Figures and Tables

**Table 1:** Criteria of clinical suspicion of VAP.

Main criteria	Sub-criteria
I. Three or more positive out of the following criteria:	1. Rectal temperature >38 °C or <35.5 °C
	2. Blood leukocytosis (>10.000/μl) and/or left shift or blood leukopenia (<3.000/μl)
	3. More than ten leukocytes in Gram stain of tracheal aspirate (in high-flow field)
	4. Positive culture of tracheal aspirate
II. New, persistent, or progressive infiltrate on chest radiograph	

**Table 2:** Characteristics of the patient groups in the study.

Characteristics	VAP(+)	VAP(−)	P-value
Sample size	32	68	
Average age [years]	64 ± 12	60 ± 14.5	0.16
Male/Female	26/6	44/24	0.07
SOFA at time of BAL	6.4 ± 3.4	6.9 ± 2.9	0.41
Severe sepsis	11 (34%)	24 (35%)	0.93
ICU mortality	12 (38%)	31 (45%)	0.45
In hospital mortality	14 (43%)	37 (54%)	0.34
Diagnostic group at admission (p-value = 0.24)			
Gastrointestinal	4 (13%)	9 (13%)	
Cardiovascular	9 (28%)	13 (19%)	
Hematologic	3 (9%)	15 (22%)	
Neurologic	4 (13%)	6 (9%)	
Orthopaedic/trauma	4 (13%)	2 (3%)	
Respiratory	8 (25%)	20 (29%)	
Other	0 (0%)	3 (4%)	
Presence of comorbidities (p-value = 0.80)			
No comorbidity	17 (53%)	38 (56%)	
One comorbidityTwo comorbidities	8 (25%)5 (16%)	15 (22%)12 (18%)	
≥Three comorbidities	2 (6%)	3 (4%)	
Distribution of comorbidities (p-value = 0.23)			
cardiovascular	3 (9%)	3 (4%)	
respiratory	3 (9%)	5 (7%)	
chronic renal failure	4 (13%)	7 (10%)	
active malignancy	2 (6%)	9 (13%)	
immunocompromised	7 (22%)	15 (22%)	
neurologic impairment	3 (9%)	8 (12%)	
chronic liver failure	2 (6%)	2 (3%)	

The age and SOFA scores were tested for significance with a two-sided paired t-test; significance was tested for the diagnostic groups using a Chi Square test. P < 0.05 was considered significant. Age and SOFA scores are represented as mean ± standard deviation.

**Table 3:** Identified VOCs for the comparison between VAP(+) and VAP(−).

Compound name	CAS nr	Molecular formula	M/z of parent molecule (g mol-1)	Average retention time (min)	Up/Down in VAP(+) vs. VAP(−)
butane, 2-methyl	78-78-4	C5H12	72.10	2.28	↑
Ethanol	64-17-5	C2H6O	46.04	2.26	↑
Acetone	67-64-1	C3H6O	58.04	2.59	↓
Isopropyl Alcohol	67-64-1	C3H8O	60.06	2.61	↓
Acrolein	107-02-8	C3H4O	56.03	2.55	↓
Furan, tetrahydro-	109-99-9	C4H8O	72.06	5.41	↓
Heptane	142-82-5	C7H16	100.13	7.25	↑
Ethylbenzene	100-41-4	C8H10	106.08	11.49	↑
Carane	17530-24-4	C10H18	138.14	14.30	↑
Dodecane	112-40-3	C12H26	170.20	17.47	↓
Tetradecane	629-59-4	C14H30	198.23	20.46	↑
Tetradecanal	124-25-4	C14H28O	212.37	23.18	↑

In addition to the compound name, CAS numbers were added for identification. Up/Down in VAP(+) vs. VAP(−) was based on mean peak height.

**Table 4:** KEGG pathways that contained one of the identified VOCs.

Compound name	KEGG pathway ID	Pathway name	Microorganisms (M), human(H), or both(B)	Produced (P), used (U) or intermediate (I)?
Ethanol	00010	Glycolysis/Gluconeogenesis	B	P
	01100	Metabolic pathways*	B	P
	01110	Biosynthesis of secondary metabolites*	M	P
	01120	Microbial metabolism in diverse environments*	M	P
	01130	Biosynthesis of antibiotics*	B	P
	04750	Inflammatory mediator regulation of TRP channels	H	U
Acrolein	00982	drug metabolism – cytochrome P450	H	P

For each pathway, the pathway ID and its pathway name as stated in the database are given, and additionally the species in which that pathway is present and whether the compound of interest is an end-product (produced), is being used (used), or is being produced as an intermediate for further utilization (intermediate). ^*^generic pathways, which contain other smaller pathways.
