# Supplementary material for: Analysis of volatile organic compounds in exhaled breath to diagnose ventilator-associated pneumonia
Source: Sci Rep. 2015 Nov 26;5:17179. doi: 10.1038/srep17179 (PMC4660425; doi:10.1038/srep17179)
Supplement: Supplementary Information [file srep17179-s1.doc]

**Analysis of volatile organic compounds in exhaled breath to diagnose ventilator-associated pneumonia**

Ronny Schnabel^1‡^, Rianne Fijten^2‡^ , Agnieszka Smolinska^2^ , Jan Dallinga^2^, Marie-Louise Boumans^3^, Ellen Stobberingh^3^, Agnes Boots^2^, Paul Roekaerts^1^, Dennis Bergmans^1^, Frederik Jan van Schooten^2*^

^1^ Department of Intensive Care Medicine, Maastricht University Medical Centre+, Maastricht, the Netherlands

^2^Department of Pharmacology and Toxicology, School for Nutrition and Translational Research in Metabolism (NUTRIM), Maastricht University Medical Centre+, Maastricht, The Netherlands

^3^ Department of Medical Microbiology, Maastricht University Medical Centre+, Maastricht, the Netherlands

^‡^ R.M. Schnabel and R.R.R. Fijten equally contributed to the manuscript and have a shared first authorship.

# Supplementary information

**Table S1: Statistical significance of confounding factors tested by rMANOVA.** The presence of one or more comorbidities was tested as well as the presence of specific comorbidities. None of the potential confounding factors were significantly associated with the VOC profile.

| **Confounder** | **p-value** |
| --- | --- |
| Age | 0.5400 |
| Gender | 0.3750 |
| SOFA score | 0.1970 |
| ICU mortality | 0.2160 |
| In hospital mortality | 0.3030 |
| Diagnostic group upon admission | 0.2370 |
| Severe sepsis | 0.2070 |
| Comorbidities (present yes/no) | 0.1790 |
| - Respiratory | 0.4090 |
| - Chronic renal failure | 0.9460 |
| - Cardiovascular | 0.1590 |
| - Active malignancy | 0.4200 |
| - Immunocompromised | 0.7040 |
| - Chronic liver failure | 0.2320 |
| - Neurological impairment | 0.3020 |
